# Concentration-Dependent Opposite Effects of 1-Benzyl-1,2,3,4-tetrahydroisoquinoline on Markers of Apoptosis: In Vitro and Ex Vivo Studies

**DOI:** 10.1007/s12640-013-9436-x

**Published:** 2013-11-05

**Authors:** Agnieszka Wąsik, Małgorzata Kajta, Tomasz Lenda, Lucyna Antkiewicz-Michaluk

**Affiliations:** 1 Department of Neurochemistry, Institute of Pharmacology Polish Academy of Sciences, 12 Smetna Street, 31-343 Kraków, Poland; 2 Department of Neuroendocrinology, Institute of Pharmacology Polish Academy of Sciences, 31-343 Kraków, Poland; 3 Department of Neuropsychopharmacology, Institute of Pharmacology Polish Academy of Sciences, 31-343 Kraków, Poland

**Keywords:** 1-Benzyl-1,2,3,4-tetrahydroisoquinoline, Apoptosis, Caspase-3, Lactate dehydrogenase, α-Synuclein, Tyrosine hydroxylase

## Abstract

1-Benzyl-1,2,3,4-tetrahydroisoquinoline (1BnTIQ) was shown to be neurotoxic to the dopaminergic neurons, and thus it was proposed to be an endogenous risk factor leading to Parkinson’s disease. In order to better understand the molecular mechanisms of 1BnTIQ—produced toxicity, we examined the impact of different concentrations of 1BnTIQ (50, 100, and 500 μM) on glutamate-induced apoptotic pathway. We measured the markers of apoptosis, such as caspase-3 activity, lactate dehydrogenase release, and mitochondrial membrane potential. Molecular data were supported at the cellular level by calcein AM and Hoechst 33342 staining. The obtained data demonstrated concentration-dependent effects of 1BnTIQ opposing apoptosis, and evidenced that 1BnTIQ in a low concentration (50 μM) exhibited neuroprotective activity, whereas in 10 times higher concentration (500 μM) might be neurotoxic, and significantly intensified glutamate-induced increase in apoptosis markers. Additionally, using an ex vivo molecular study we indicated that both acute and chronic administration of 1BnTIQ did not affect the level of alpha synuclein and tyrosine hydroxylase protein in the rat substantia nigra. Summarizing the studies, we suggest that 1BnTIQ is a rather weak endogenous neurotoxin; however, it should be taken into account that in higher μmoles concentrations, it can initiate apoptosis in the central nervous system and may be involved in the etiopathology of neurodegenerative diseases.

## Introduction

The programed cell death (apoptosis) is a biological process in which unwanted and/or damaged cells are killed, so that the organism could maintain homeostasis. The apoptosis process is a characteristic not only of normal development but also of numerous neurodegenerative diseases and neurological conditions, such as Parkinson’s disease (PD), Alzheimer’s disease (AD), Huntington’s disease, and stroke (Bamberger and Landreth 2002; Jenner 2003; Mattson 2003; McLaughlin et al. 1998; Morishima et al. 2001). Keep in mind that in the neurodegenerative diseases, neuronal cells death occurs not only via apoptosis but also in the necrosis. Apoptosis mainly affects the cell nucleus and is related to specific DNA fragmentation which results in apoptotic body formation and individual cell death without inflammatory response to damage (Honda et al. 2001). Apoptotic cells may be characterized by specific morphological and biochemical changes, including cell shrinkage, chromatin condensation, and internucleosomal cleavage of genomic DNA (Kajta et al. 2004). At the molecular level, apoptosis is regulated by the activation of the caspase cascade, which depends on nonspecific insults leading to calcium influx into the cytoplasm and reduction of mitochondrial membrane potential (internal pathway). Alternative apoptotic pathway (external pathway) is mediated through death receptors (Kajta et al. 2004). Caspase-3 has been proposed to be a crucial executioner protease of the apoptotic cascade (Kuida et al. 1996; Pompeiano et al. 2000). Although apoptosis is usually a caspase-dependent process, it may also be unrelated to caspases, but mediated by other enzymes, such as calpains, which accompany programed cell death during neuronal degeneration.

A number of in vitro studies have demonstrated that glutamate is a potent neurotoxin capable of destroying neurons by apoptosis (Froissard and Duval 1994; Behl et al. 1995; Kajta et al. 2007, 2013). In this way, glutamate represents a good model for the study of apoptosis. In primary cortical cells, glutamate-induced cell death involves upregulation of caspase-3 and its activation via the caspase-dependent pathway involving mitochondrial signaling (Zhang and Bhavnani 2005). Possible factors responsible for the neuron degeneration include: oxidative stress, mitochondrial dysfunctions, protein mishandling, apoptosis, and inflammation (Gandhi and Wood 2005; Moore et al. 2005; Sas et al. 2007). Dopaminergic brain structures are particularly sensitive to oxidative stress, because the dopamine metabolism itself leads to generation of reactive oxygen species (ROS). ROS generation initiates mitochondrial-caspase cascade which leads to the activation of the main effector of apoptosis, i.e., caspase-3 (Hanrott et al. 2006; Bayir et al. 2009).

1-Benzyl-1,2,3,4-tetrahydroisoquinoline (1BnTIQ) is an endogenous neurotoxin which has been proposed to be one of the etiological factors of idiopathic PD (Kotake et al. 1995). The concentration of 1BnTIQ in the CSF of parkinsonian patients was three times higher than in the CSF of neurological control subjects (Kotake et al. 1995). Chronic treatment with 1BnTIQ produced parkinsonian-like symptoms in rodents and primates (Kotake et al. 1995, 1996). Some evidence demonstrated that 1BnTIQ-induced cell death via apoptosis, and dose dependently elevated the level of the pro-apoptotic protein Bax and decreased the concentration of the antiapoptotic protein Bcl-xl. Additionally, 1BnTIQ increased the formation of the active caspase-3 protein fragments (Shavali and Ebadi 2003). 1BnTIQ, which is synthesized endogenously in the brain and/or is obtained exogenously in the diet, can be taken up by neurons via DAT. Furthermore, it accumulates in the dopaminergic neurons and may exert some pathological effects leading to parkinsonism (Okada et al. 1998). As previously demonstrated, 1BnTIQ strongly affected dopamine metabolism, and similar to the other exogenous neurotoxins potentiated MAO-dependent dopamine oxidation and inhibited COMT-dependent O-methylation catabolic pathways (Antkiewicz-Michaluk et al. 2001; Wąsik et al. 2009). What is more, 1BnTIQ might inhibit monoamine vesicular transporter2 (VMAT2), and led to an increase of the oxidative stress by generation of ROS in dopaminergic neurons (Wąsik et al. 2009). In the light of these data, the question arises whether neurotoxic effect of 1BnTIQ is mainly observed in dopaminergic structures? It was also interesting to examine whether in vitro effects correspond with the ex vivo experiments.

The aim of the present study was a systematic analysis of the effects of different concentrations of 1BnTIQ on apoptosis markers in in vitro models as well as after its in vivo administration.

## Materials and Methods

### Animals and Treatment

The ex vivo experiments were carried out on male Wistar rats whose initial body weight was 220–240 g. All animals had free access to standard laboratory food and tap water, and were maintained at room temperature (RT) (22 °C) under an artificial light/dark cycle (12/12 h, light on at 7 a.m.).

The rats were administered 1BnTIQ at a dose of 50 mg/kg intraperitoneally (ip*)* once or chronically for 14 consecutive days. Control rats were treated with an appropriate solvent. The rats were killed by decapitation 3 h (caspase-3 activity) or 24 h (α-synuclein and tyrosine hydroxylase) after the last drug injection, and different structures of the brain were dissected. The experiments were carried out between 9.00 and 16.00 h.

All procedures were carried out in accordance with the National Institutes of Health Guide for the Care and Use of Laboratory Animals and were granted an approval from the Bioethics Commission as compliant with Polish Law. All the experimental procedures were approved by the Local Bioethics Commission of the Institute of Pharmacology, Polish Academy of Sciences in Kraków.

### Drugs

1BnTIQ hydrochloride was synthesized (according to Cannon and Webster, 1958) at the Department of Drug Chemistry of the Institute of Pharmacology, the Polish Academy of Sciences in Kraków. Purity of the compound was verified by measurement of its melting point; and homogeneity was assessed on a chromatographic column. The compounds were dissolved in a 0.9 % NaCl solution. Glutamic acid was purchased from Sigma-Aldrich (St. Louis, MO, USA), whereas calcein AM and Hoechst 33342 were purchased from Molecular Probes (Eugene, OR, USA).

#### Primary Hippocampal Cell Cultures

Hippocampal tissues for primary cultures were prepared from Wistar rat or Swiss mouse embryos (Charles River, Germany) at 15th–17th day of gestation and were cultured essentially as described earlier (Junghans and Kappler 1999; Kajta et al. 2009). Animal care followed official governmental guidelines, and all efforts were made to minimize the number of animals used and their suffering. All procedures were carried out in accordance with the National Institutes of Health Guidelines for the Care and Use of Laboratory Animals, and were granted an approval from the Bioethics Commission as compliant with Polish Law (21 August 1997). The cells were suspended in estrogen-free neurobasal medium supplemented with B27 and plated at a density of 2.5 × 10^5^ cells per cm^2^ onto poly-ornithine (0.01 mg/ml)-coated multiwell plates. The cultures were maintained at 37 °C in a humidified atmosphere containing 5 % CO_2_ for 7 days in vitro (DIV) prior to experimentation. The level of astrocytes, as determined by the content of intermediate filament protein GFAP (glial fibrillary acidic protein), did not exceed 10 % for all cultures (Kajta et al. 2004).

#### Treatment

Primary hippocampal cell cultures were exposed to glutamate (1 mM), 1BnTIQ (50, 100, 500 μM), or glutamate and 1BTIQ for 24 h. To avoid unspecific effects in our study, 1BnTIQ was used in the concentrations which did not affect the control level of caspase-3 activity and lactate dehydrogenase (LDH) release. All the compounds were originally dissolved in dimethyl sulfoxide (DMSO), and then further diluted in culture medium to achieve DMSO concentrations below 0.1 %.

#### Identification of Apoptotic Cells

Apoptotic cells were detected by Hoechst 33342-staining 24 h after initial treatment, as described previously (Kajta et al. 2013). Hippocampal cells cultured on glass cover slips were washed with 10 mM phosphate-buffered saline (PBS) and exposed to Hoechst 33342 (0.6 mg/ml), at RT for 5 min. Cells with bright blue-fragmented nuclei, showing condensed chromatin, were identified as apoptotic cells. Qualitative analysis was performed using a fluorescence microscope (Leica Microsystems Wetzlar GmbH, Wetzlar, Germany) connected to a CoolSnap camera (Vision Systems GmbH, Puchheim, Germany) with MetaMorph software.

#### Staining with Calcein AM

Staining with calcein AM was based on intracellular esterase activity in hippocampal cultures 24 h after initial treatment (Kajta et al. 2013). In order to block esterase activity present in the growth media, cells were washed with PBS. The cells grown on glass cover slips were then incubated in 2 μM calcein AM in PBS at RT for 10 min. Cells with bright yellow cytoplasm were identified as living cells. A fluorescence microscope (Leica Microsystems Wetzlar GmbH, Wetzlar, Germany) connected to a CoolSnap camera (Vision Systems GmbH, Puchheim, Germany) with MetaMorph software was used for qualitative analyses.

#### Assessment of Mitochondrial Membrane Potential

The mitochondrial membrane potential was determined with the JC-1 Assay Kit, which utilizes a cationic dye 5,5′,6,6′-tetrachloro-1,1′,3,3′-tetraethylbenzimidazolylcarbo-cyanine iodide. In healthy cells, the dye aggregates and stains the mitochondria bright red, whereas in apoptotic cells, the mitochondrial membrane potential collapses, and the dye remains in the cytoplasm in a green fluorescent monomeric form (Hirsch et al. 1998). The assessment of the loss of mitochondrial membrane potential, which is a hallmark of apoptosis, was performed in the hippocampal cultures treated for 6 h with glutamic acid (1 mM) and 1BnTIQ (50, 500 μM) alone or in combination. Cells were incubated with JC-1 solution for 25 min, and red (550/600 nm) and green (485/535 nm) fluorescence were measured with an Infinite M1000 microplate reader (Tecan, Austria). The data were analyzed with I-control software, normalized to the fluorescence in vehicle-treated cells, and expressed as the red-to-green fluorescence ratio ± SEM of three to four independent experiments. The fluorescence of blanks, acting as no enzyme controls, was subtracted from each value.

#### Assessment of Caspase-3 Activity

Caspase-3 activity was determined according to Nicholson et al. (1995), using samples treated for 24 h with glutamate (1 mM) alone or in combination with the test compound. The assessment of caspase-3 activity was performed as previously described (Kajta et al. 2009). Cell lysates were incubated at 36 °C with a colorimetric substrate that is preferentially cleaved by caspase-3, called Ac-DEVD-*p*NA (*N*-acetyl-asp-glu-val-asp-*p*-nitro-anilide). The amounts of *p*-nitroanilide were monitored continuously over 60 min with a Multiskan Spectrum Microplate Spectrophotometer (ThermoLabsystems, Vantaa, Finland). Data were analyzed with Ascent software, normalized to the absorbance in vehicle-treated cells, and expressed as percent of control ± SEM of three to four independent experiments. The absorbance of blanks, acting as no enzyme controls, was subtracted from each value.

#### Measurement of Lactate Dehydrogenase Activity

To quantify cell death, LDH released from damaged cells into the cell culture media was measured 24 h after treatment with glutamate and 1BnTIQ (50, 100, 500 μM). LDH release was measured as previously described (Kajta et al. 2004). Cell-free culture supernatants were collected from each well and incubated with the appropriate reagent mixture according to the supplier’s instructions (Cytotoxicity Detection Kit) at RT for 30–60 min depending on reaction progress. The intensity of red color formed in the assay, measured at a wavelength of 490 nm, was proportional to both LDH activity and the number of damaged cells. Data were normalized to the color intensity from vehicle-treated cells (100 %) and expressed as percent of control of three to four independent experiments. The total LDH release was determined in the cell cultures treated with 1 % Triton X-100 for 24 h. The total LDH release reached value of 1.100 ± 0.190 U/h/100 μg protein. In control cultures, the absolute value of LDH activity was 0.300 ± 0.050 U/h/100 g protein and was similar to that obtained by Mytilineou et al. (1998).

#### Western Blot Analysis of α-Synuclein (αSyn) and Tyrosine Hydroxylase (TH) Protein

Isolated structures (substantia nigra and striatum) originating from 1-BnTIQ or solvent-treated rats were weighted, homogenized on ice in 20 volumes of RIPA buffer (150 mM NaCl, 1.0 % NP-40, 0.5 % sodium deoxycholate, 0.1 % SDS, 50 mM Tris, pH 8.0) with addition of protease inhibitors mixture (Pierce). Protein concentration in the supernatants was determined using bicinchoninic acid protein assay kit (Pierce). Afterward, the samples containing 5 μg of total protein were fractionated by 12 or 10 % sodium dodecyl sulfate–polyacrylamide gel electrophoresis (SDS-PAGE), as described previously by Laemmli (1970) and processed to detect α-synuclein or tyrosine hydroxylase. Proteins from the resolved gels were then transferred to nitrocellulose membranes (Sigma). Nonspecific binding sites were blocked overnight at 4 °C by a 3 % BSA in Tris-buffered saline with a 0.5 % Tween 20 (TBS-T) and incubated for 2 h with a mouse monoclonal anti-αSyn antibody (BD Transduction Laboratories, dilution 1:2000) or mouse monoclonal anti-TH antibody (Millipore, dilution 1:4000) in 1 % BSA at RT. After three washes in TBS-T, membranes were processed according to the standard BM Chemiluminescence Western Blotting Kit protocol (Roche Applied Science). Following immunoblot visualization, membranes were blocked with a 5 % nonfat dry milk in TBS for 10 min at RT, and dried on absorbent filter paper. Afterward, blots were erased in 62.5 mM TrisCl pH 6.8, 2 % SDS, 100 mM 2-mercaptoethanol for 30 min at 50 °C, washed twice with TBS and blocked overnight with a 5 % nonfat dry milk in TBS at 4 °C. As a control to protein level normalization, erased blots were processed with mouse monoclonal anti-β-actin antibody (Santa Cruz Biotechnology, Inc., dilution 1:1000), as described above.

The amounts of protein per lane as well as antibody concentrations were optimized in pilot studies so that at least threefold differences in protein content were linearly reflected on immunoblots.

The signals were visualized and quantified by the densitometric analysis with the FUJI-LAS 4000 system and Fuji Multi Gauge software. Results are presented as a percentage of the control of the analyzed protein: β-actin ratio ± SEM.

#### Calculations and Statistics

Statistical tests were performed on raw data expressed as the mean arbitrary absorbance or fluorescence units per well containing 50,000 cells (measurements of caspase-3, LDH, mitochondrial potential). A one-way analysis of variance (ANOVA) was used to determine overall significance. Differences between control and experimental groups were assessed with the post hoc Neuman–Keuls test, with the significant differences marked in the following way: *p < 0.05; **p < 0.01; ***p < 0.001 (vs control group); ^#^ *p* < 0.05; ^##^ *p* < 0.01; ^###^ *p* < 0.001 (vs glutamate group).

Statistical significance of the data from Western blot analysis was assessed using a one-way ANOVA followed (if significant) by the Tukey test for post hoc comparison with the control (saline) group.

## Results

### The Effects of 1BnTIQ on Glutamate-Induced Caspase-3 Activity and LDH Release in Hippocampal Cultures

**Fig. 1:**
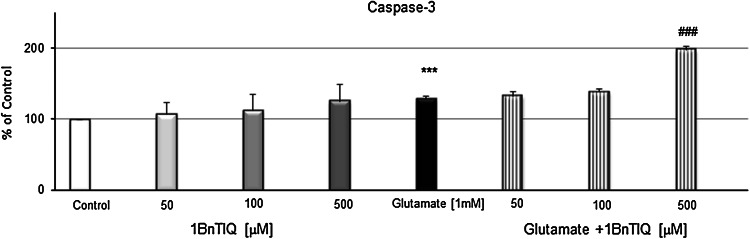
The effect of 1BnTIQ on glutamate-induced caspase-3 activity in rat hippocampal cultures. Cells were treated either with glutamate (1 mM) or 1BnTIQ (50, 100 or 500 μM) alone or in combination. The results are presented as a percentage of control. Each *bar* represents the mean of three to four independent experiments ± SEM. The number of replicantes in each experiment ranged from 5 to 8. ***p < 0.001 versus control cultures; ^###^ *p* < 0.001 versus the cultures exposed to glutamate

In hippocampal cultures exposed to 1 mM glutamate for 24 h, the activity of caspase-3 increased by 30 % (*p* < 0.001) (Fig. 1). Similar effect was observed in the presence of glutamate and 1BnTIQ in the low concentrations (50 and 100 μM). In contrast, the highest concentration of 1BnTIQ (500 μM) significantly (*p* < 0.001) intensified the effect of glutamate and produced elevation of caspase-3 activity by 100 % (Fig. 1).

**Fig. 2:**
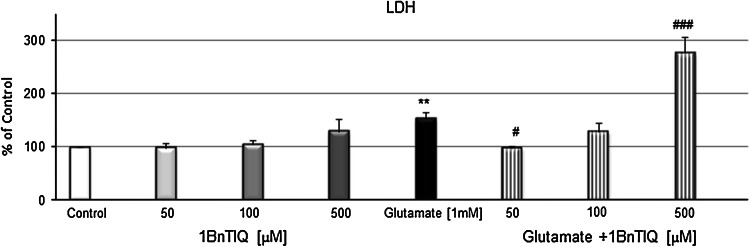
The effect of 1BnTIQ on glutamate-induced release of LDH in rat hippocampal cultures. Cells were treated either with glutamate (1 mM) or 1BnTIQ (50, 100 or 500 μM) alone or in combination. The results are presented as a percentage of control. Each *bar* represents the mean of three to four independent experiments ± SEM. The number of replicantes in each experiment ranged from 5 to 8. **p < 0.01 versus control cultures; ^*#*^ *p* < 0.05; ^###^ *p* < 0.001 versus the cultures exposed to glutamate

LDH release increased within the duration of glutamate treatment by 30 % (*p* < 0.01) (Fig. 2). 1BnTIQ in the lower concentration (50 μM) inhibited glutamate-induced LDH release, to the control level. 1BnTIQ in the middle concentration (100 μM) was not active, whereas its highest concentration (500 μM) significantly (*p* < 0.001) intensified glutamate-induced release of LDH by 190 % (Fig. 2).

In the cells treated with 50 and 100 μM 1BnTIQ, the activity of caspase-3 was ~110 % (Fig. 1), and LDH release reached the average value of 101 % (Fig. 2). Exposure to 500 μM 1BnTIQ produced visible (~130 %), but also not significant increase in the levels of caspase-3 (Fig. 1) and LDH (Fig. 2).

### The Influence of 1BnTIQ on Glutamate-Induced Loss of Mitochondrial Membrane Potential in Mouse Hippocampal Cultures

**Fig. 3:**
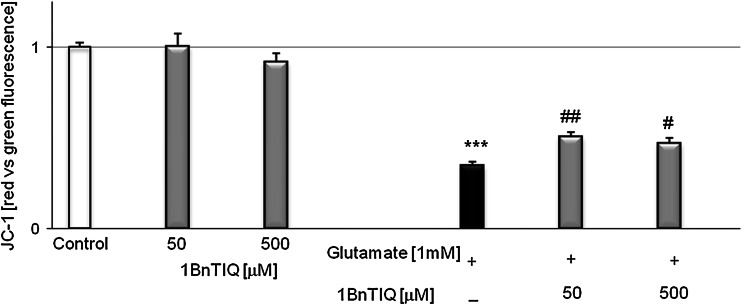
The influence of 1BnTIQ on glutamate-induced loss of mitochondrial membrane potential in mouse hippocampal cultures. Primary hippocampal cultures were treated with glutamate (1 mM) or 1BnTIQ (50 or 500 μM) alone or in combination, for 6 h. The mitochondrial membrane potential was detected with JC-1 Assay Kit. The results were normalized to the fluorescence in vehicle-treated cells and expressed as *red* to *green* fluorescence ratio. Each *bar* represents the mean of three to four independent experiments ± SEM. The number of replicantes in each experiment ranged from 5 to 8. ***p < 0.001 versus control cultures; ^*#*^ *p* < 0.05; ^*##*^ *p* < 0.01 versus the cultures exposed to glutamate

6 h exposure of hippocampal cultures to glutamate (1 mM) caused an about 70 % loss of mitochondrial membrane potential in the neuronal cells (*p* < 0.001) (Fig. 3). Co-treatment with 1BnTIQ in concentrations of 50 and 500 μM partially reversed this effect increasing the mitochondrial membrane potential to ~50 % of the control value (Fig. 3). The administration of 1BnTIQ in both used concentrations (50 and 500 μM) alone did not affect the nonstimulated control levels of the measured parameters (Fig. 3).

### The Influence of 1BnTIQ on Glutamate-Induced Changes in Calcein AM and Hoechst 33342 Staining in Hippocampal Cultures

**Fig. 4:**
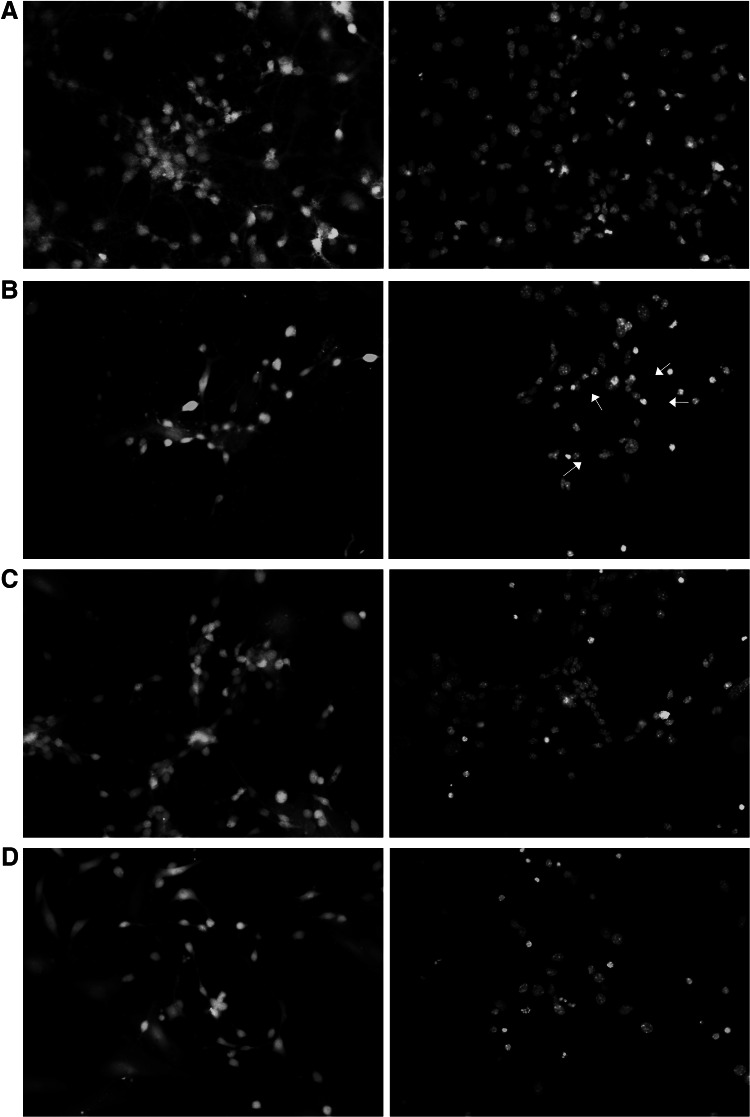
The influence of 1BnTIQ (50 or 500 μM) on glutamate-induced (1 mM) changes in calcein AM (*first column*) and Hoechst 33342 (*second column*) staining in rat hippocampal cultures, examined 24 h post-treatment. **a** Control, **b** glutamate (1 mM), **c** Glutamate (1 mM) + 1BnTIQ (50 μM), **d** Glutamate (1 mM) + 1BnTIQ (500 μM). Cells were cultured on glass cover slips, washed with 10 mM PBS, and exposed to 2 μM calcein AM at RT for 10 min. Cells were then rewashed and incubated with Hoechst 33342 (0.6 μg/ml) at RT for 5 min. Cells with bright fragmented nuclei showing condensed chromatin (see *arrow*s) were identified as undergoing apoptosis, whereas cells with *light*-*colored* cytoplasm were identified as living cells. Hoechst 33342 stain is a cell-permeant nuclear counterstain that emits blue fluorescence when bound to dsDNA. This dye is often used to distinguish condensed pycnotic nuclei (marked by *arrows*) in apoptotic cells (Color figure online)

A continuous 24-h exposure of hippocampal cultures to glutamate (1 mM) reduced the density of living cells, as indicated by the decreased number of cells with light-colored cytoplasm (Fig. 4b). Treatment with glutamate substantially enhanced the number of bright fragmented nuclei with condensed chromatin, which is typical of cells undergoing apoptosis (Fig. 4b).

Co-treatment with 1BnTIQ at 50 μM normalized the number of healthy living cells and diminished the number of fragmented nuclei (Fig. 4c). In contrast to this, a higher concentration of 1BnTIQ (500 μM) did not affect on glutamate-induced changes in calcein AM and Hoechst 33342 staining in the hippocampal cultures (Fig. 4d).

### The Effect of Acute and Chronic Administration of 1BnTIQ on Caspase-3 Activity in the Rat Hippocampus. An Ex Vivo Study

**Fig. 5:**
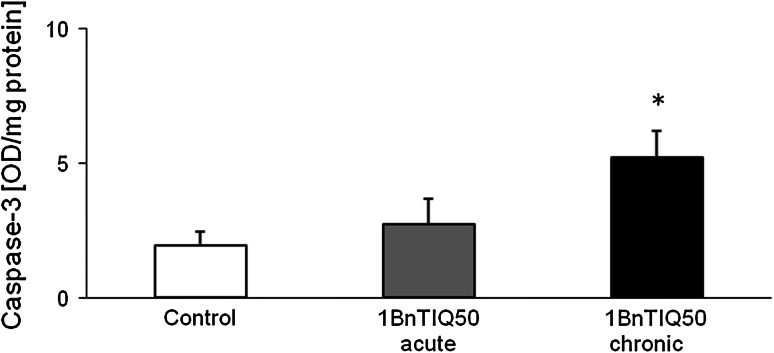
The effect of acute and chronic administration of 1BnTIQ on caspase-3 activity in the rat hippocampus. An ex vivo study: 1BnTIQ was administered acute (×1) or chronic at dose 50 mg/kg i.p. during 14 consecutive days. The control group was treated with saline. The rats were decapitated 3 h after last injection. The results are expressed as the mean ± SEM of six samples (*n* = 6 animals per group). Data were analyzed by means of one-way ANOVA followed by Neuman–Keuls test. Statistical significance *p < 0.05 versus Saline group

Acute systemic administration of 1BnTIQ in a dose of 50 mg/kg produced only a slight elevation of the caspase-3 activity, whereas chronic (14 days) administration of 1BnTIQ induced a significant (*p* < 0.05) increase in this parameter (by ~90 %) (Fig. 5).

### The Effects of Acute and Chronic 1BnTIQ Administration on the α-Synuclein Level in the Rat Substantia Nigra as Measured 24 h After the Last Dose. An Ex Vivo Study

**Fig. 6:**
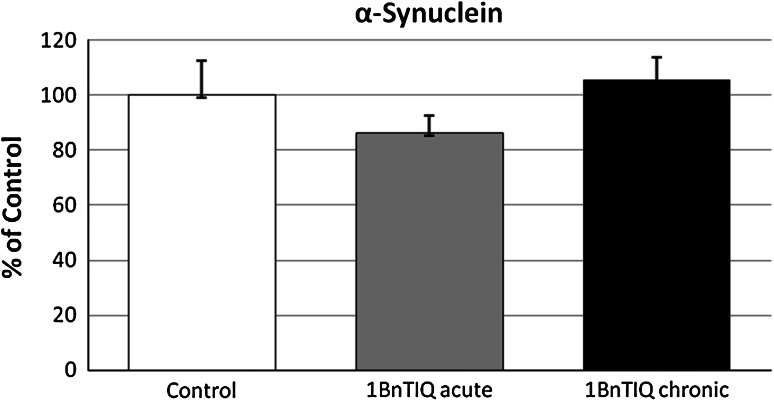
The effects of acute and chronic 1BnTIQ administration on the alpha-synuclein level in the substantia nigra, 24 h withdrawal. 1BnTIQ was administered acute or chronic at dose 50 mg/kg i.p. during 14 consecutive days. The control group was treated with saline. The rats were decapitated 24 h after last injection. The results are expressed as the mean ± SEM of six samples (*n* = 6 animals per group). Data were analyzed by means of one-way ANOVA followed by Tukey test. Statistical significance *p < 0.05; **p < 0.01 versus Saline group

The data in Fig. 6 demonstrate that both acute and chronic (14 days) treatment with 1BnTIQ (50 mg/kg i.p.) did not change the level of α-synuclein in the substantia nigra measured 24 h after the last dose.

### The Effects of Acute and Chronic 1BnTIQ Administration on the Tyrosine Hydroxylase Level in the Substantia Nigra as Measured 24 h After the Last Dose. An Ex Vivo Study

**Fig. 7:**
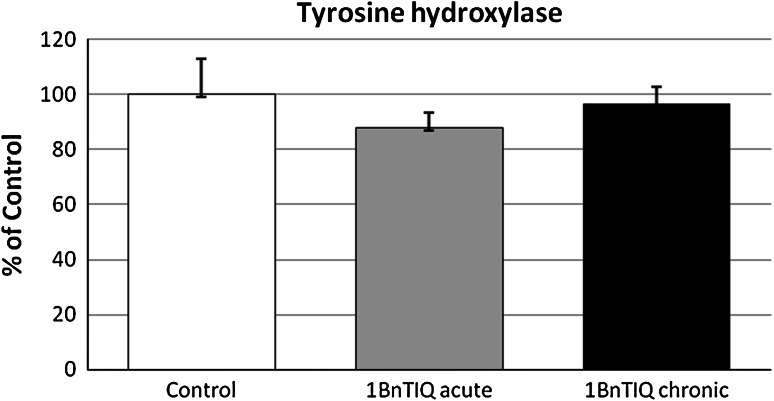
The effects of acute and chronic 1BnTIQ administration on the tyrosine hydroxylase level in the substantia nigra, 24 h withdrawal. 1BnTIQ was administered acute or chronic at dose 50 mg/kg i.p. during 14 consecutive days. The control group was treated with saline. The rats were decapitated 24 h after last injection. The results are expressed as the mean ± SEM of six samples (*n* = 6 animals per group). Data were analyzed by means of one-way ANOVA followed by Tukey test. Statistical significance *p < 0.05; **p < 0.01 versus Saline group

Both acute and chronic 14-day administration of 1BnTIQ (50 mg/kg i.p.) did not change the level of tyrosine hydroxylase in the substantia nigra measured 24 h after the last does (Fig. 7).

## Discussion

The main finding of this paper is that 1BnTIQ produced concentration-dependent opposite effects on markers of apoptosis evaluated by in vitro studies in primary hippocampal cultures of the mouse and the rat. 1BnTIQ in a low concentration (50 μM) possessed neuroprotective activity, while in a 10 times higher concentration (500 μM) might be neurotoxic, and significantly intensified glutamate-induced neurotoxicity. On the other hand, in vivo chronic administration of 1BnTIQ did not produce neurotoxic effects on dopamine neurons in the substantia nigra as assessed by the levels of two proteins: tyrosine hydroxylase and alpha-synuclein.

1BnTIQ is an endogenous neurotoxin which has been proposed to be an etiological factor of PD (Kotake et al. 1995; Antkiewicz-Michaluk et al. 2001). 1BnTIQ can be formed in vivo in the mammalian brain from phenylethylamine and phenylacetaldehyde. This compound belongs to the isoquinoline group and its structure closely resembles the well-known exogenous toxin, MPTP; however, the potency and mode of action of both these substances are completely different. While MPTP acts rapidly and produces irreversible neurotoxic changes even after a single injection (Burns et al. 1985), 1BnTIQ produces a nonimmediate and milder neurotoxic effect (Antkiewicz-Michaluk et al. 2000, 2001; Lorenc-Koci et al. 2004). In the light of these observations, 1BnTIQ may offer a better model of PD, exhibiting slowly developing neurodegenerative changes. In fact, the level of 1BnTIQ in the CSF has been reported to be three times higher in PD patients than in control subjects (Kotake et al. 1995).

In the present study, we used the glutamate model of neurotoxicity to induce apoptosis. A number of in vitro studies revealed that a high concentration (mM) of glutamate destroyed neurons (Froissard and Duval 1994; Davis and Maher 19933). Glutamate toxicity involves oxidative stress and apoptosis (Coyle and Puttfarcken 1993; Nicotera et al. 1997). Exposure to glutamate has been associated with an increase in cytosolic Ca^2+^ in the cells (Atlante et al. 2001). Additionally, a long exposure to glutamate resulted in permanent damage of mitochondria, which occurred simultaneously with a high mitochondrial ROS production (Beal et al. 1997). As shown in this paper, glutamate in mM concentration significantly elevated the caspase-3 activity (Fig. 1). In the combined treatment groups, only the highest concentration of 1BnTIQ (500 μM) produced potentiation of the glutamate effect on caspase-3 activity (Fig. 1) and LDH release (Fig. 2) in the rat hippocampal cultures. Lower concentrations of 1BnTIQ (50 and 100 μM) did not potentiate the toxic effect of glutamate or even inhibited the toxicity in the rat hippocampus, leading to reduction in the LDH release (Fig. 2). The results presented in this paper suggest that in the primary cultures of rodent hippocampus 1BnTIQ may act differently depending on the applied concentration: showing neuroprotective properties in a lower concentration and neurotoxic activity in higher concentrations. It should be mentioned that in contrast to our studies, a majority of authors investigated the biochemical and molecular mechanisms of neurotoxicity of 1BnTIQ mainly in dopaminergic cells or structures (Kotake et al. 1995; Shavali and Ebadi 2003; Wąsik et al. 2009). Now, for the first time, we have found that 1BnTIQ can destroy nondopaminergic structures, e.g., hippocampus. The hippocampus is composed of different kinds of neurons, viz. it contains a high proportion of glutamatergic neurons and small amount of dopaminergic neurons. For this reason, the hippocampus is a very sensitive structure to the toxic effects of glutamate and is a good model for investigation of apoptosis. Previously, different authors who studied the process of apoptosis have focused mainly on the dopaminergic system.

Shavali et al. (2004) observed toxic effects of 1BnTIQ even at low concentrations when they were evaluated in human dopaminergic SH-SY5Y cells. The authors demonstrated that lower concentrations of 1BnTIQ (1–100 μM) produced a decrease in reduced glutathione (GSH) and an increase in alpha-synuclein levels, whereas higher concentrations of 1BnTIQ (250 and 500 μM) significantly elevated ROS levels and decreased ATP content. It is interesting that in human dopaminergic SH-SY5Y cells, a protective effect of a low concentrations of 1BnTIQ was not observed. What is more, our present in vitro data indicated that 1BnTIQ used in a broad range of concentrations (50 up to 500 μM) in primary hippocampal cultures did not affect the mitochondrial membrane potential, but partially antagonized glutamate-induced loss of mitochondrial membrane potential (Fig. 3). It appears that such discrepancies between our present results and other authors’ data may be due to different study material where the proapoptotic effect of 1BnTIQ was examined (human dopaminergic SH-SY5Y cells and primary hippocampal cultures). Our present in vitro studies performed on hippocampal primary cultures of both mice and rats clearly indicate that neurotoxic action of high concentrations of 1BnTIQ occurs via apoptosis. Beyond the assessment of caspase-3 activity, we also evaluated other markers of apoptosis, since we performed the identification of apoptotic cells by Hoechst 33342-staining and by calcein AM staining in rat hippocampal cultures. We investigated the effect of 1BnTIQ in two different concentrations: 50 and 500 μM on glutamate-induced (1 mM) pro-apoptotic action. The results seem agree with the above data for caspase-3 activity and LDH release. 1BnTIQ in a lower concentration (50 μM) partially inhibited and in a higher dose (500 μM) intensified glutamate-induced pro-apoptotic effect on hippocampal cells (Fig. 4c, d).

In our ex vivo study, we obtained further interesting data on the mechanism of neurotoxic action of 1BnTIQ after its chronic in vivo administration in the rat. The results demonstrated that chronic (14 days) but not acute administration of 1BnTIQ (50 mg/kg i.p.) produced a significant elevation of caspase-3 activity in the rat hippocampus (Fig. 5), but did not change the level of alpha synuclein (Fig. 6) or tyrosine hydroxylase (Fig. 7) protein in the rat substantia nigra. This effect may be caused by low toxicity of 1BnTIQ or compensatory mechanisms such as an increase in the expression of TH in the cells survival.

The present data confirm our earlier suggestion that 1BnTIQ is an endogenous neurotoxin with a rather weak toxicity (Wąsik et al. 2009). Only a long-term impact of this compound or its high concentrations may cause damage of nerve cells. Some authors indicated that 1BnTIQ dose dependently elevated the level of the pro-apoptotic protein Bax and simultaneously decreased the concentration of the antiapoptotic protein Bcl-xl (Shavali and Ebadi 2003). A morphological analysis of SH-SY5Y cells treated with 1BnTIQ showed nuclear defects and the presence of apoptotic-like bodies and nuclear fragments (Shavali et al. 2004). Additionally, Kotake et al. (2003) demonstrated that prolonged exposure of dopaminergic neurons to a low concentration of 1BnTIQ initially induced a decrease in the dopamine level, after which the shrinkage of the cell body led to cell death. These data are in agreement with our results, in the context of 1BnTIQ neurotoxicity. Some authors observed that 1BnTIQ caused a significant elevation of the LDH release only in slices containing mainly dopaminergic neurons (mesencephalon cultures as well as co-cultures of mesencephalon and striatum), but in other regions, the differences were not significant (Kotake et al. 2003). There is a high probability that the differences between our results compared with other authors’ papers are due to a low percentage of dopaminergic neurons in the hippocampal cultures. In the light of those data and taken into account our present results, we may suggest that 1BnTIQ in a high concentration leads to cell death via apoptotic route, but may be more toxic for dopaminergic cells.

In conclusion, our study strongly indicates that 1BnTIQ in primary hippocampal cultures may exert opposite effects on markers of apoptosis depending upon its concentration: neuroprotection at low concentrations or pro-apoptotic effect at higher concentrations. However, 1BnTIQ in a high concentration and upon prolonged exposure leads to apoptotic cell death and can be one of the etiological factors of neurodegenerative disease.
